# Overexpression of YKL-40 Predicts Plaque Instability in Carotid Atherosclerosis with CagA-Positive Helicobacter Pylori Infection

**DOI:** 10.1371/journal.pone.0059996

**Published:** 2013-04-03

**Authors:** Yina Wu, Zhen Tao, Chao Song, Qingshuai Jia, Jun Bai, Kangkang Zhi, Lefeng Qu

**Affiliations:** 1 Department of Neurosurgery, Changhai Hospital, Second Military Medical University, Shanghai, China; 2 Department of Neurology, General Hospital of Jinan Military Command, Jinan, Shandong, China; 3 Department of Vascular Surgery, Changhai Hospital, Second Military Medical University, Shanghai, China; 4 Center for Disease Control and Prevention of Jinan Military Command, Jinan, Shandong, China; 5 Department of Vascular and Endovascular Surgery, Changzheng Hospital, Second Military Medical University, Shanghai, China; Campus Bio-Medico University, Italy

## Abstract

**Objectives:**

YKL-40 has been demonstrated to be related to atherosclerosis, but its role in predicting plaque status and the outcome of carotid atherosclerosis (CAS) caused by CagA-positive helicobacter pylori remains unclear. This study was aimed to investigate the role of YKL-40 in predicting the outcome of carotid atherosclerosis with CagA-positive Helicobacter pylori infection.

**Methods:**

The serum concentrations of YKL-40, C-reaction protein in 310 patients undergoing color Duplex assessment of carotid atherosclerosis were recorded and divided into 3 groups according to the infectious statuses of helicobacter pylori. We also examined serum YKL-40, C-reaction protein and the plaque morphology in animal model of carotid atherosclerosis with different types of helicobacter pylori infection.

**Results:**

Overexpression of YKL-40 was only found in carotid atherosclerosis group with CagA-positive helicobacter pylori infection; C-reaction protein failed to distinguish different infectious statuses of helicobacter pylori infection. In patients with CagA-positive helicobacter pylori infection, elevated YKL-40 expression was accompanied by more severe clinical symptoms. We also confirmed similar findings in rabbit model of carotid atherosclerosis with CagA-positive helicobacter pylori infection. We found that in 7 rabbits treated with anti-helicobacter pylori therapy, the serum YKL-40 level decreased and the plaque became more stable.

**Conclusion:**

Our findings suggested that increased serum YKL-40 level indicates plaque instability and more severe clinical symptoms of carotid atherosclerosis with CagA-positive helicobacter pylori infection. Compared with C-reaction protein, YKL-40 seems to be a more specific predictor of plaque status and outcome of carotid atherosclerosis with CagA-positive helicobacter pylori infection.

## Introduction

Atherosclerotic stroke is an increasingly critical problem worldwide. A recent study has demonstrated that CagA-positive Helicobacter pylori (CagA^+^ HP) infection is correlated with atherosclerotic stroke [1], and a further study has confirmed the relationship between CagA^+^ HP infection and the plaque instability [2]. Considering the high frequency of cerebral symptoms and hospitalization and poor prognosis of atherosclerotic stroke, patients with carotid atherosclerosis need to be followed up closely, especially those with CagA^+^ HP infection. Therefore, a novel biomarker is urgently needed to predict unstable carotid plaque and future atherosclerotic stroke risk.

The acute-phase protein YKL-40, also called as human cartilage glycoprotein-39, expressed by macrophages in late phases of differentiation [3], is a novel biomarker of inflammation. Other sources of YKL-40 include vascular smooth muscle cells and neutrophils [4], [5]. YKL-40 has been shown to play a role in the pathogenesis of endothelial dysfunction, atherosclerosis and abnormal angiogenesis [6]. Recent studies have showed that serum YKL-40 level is increased in patients with stable coronary artery disease [7]–[9], and is associated with atherosclerotic progression [10].

Our aim is to evaluate whether YKL-40 can serve as a prognostic biomarker for patients with CAS with CagA^+^ HP infection and a new biomarker for monitoring the treatment.

## Materials and Methods

### Patients and Participants

From January 2008 to October 2008, all patients that were referred to Changhai Hospital for color Duplex assessment of carotid atherosclerosis were screened for enrollment in this study.

Inclusion criteria included:

1. No evidence of atherosclerosis in other arteries including coronary artery disease and peripheral arteries;
2. No evidence of potential risk of cardiogenic thrombosis including fibrillation atrial, rheumatic heart disease;
3. No evidence of deep vein thrombosis (DVT);

Exclusion criteria included:

1. Ongoing inflammatory disease such as autoimmune disease and infection;
2. Malignant tumors;

Informed consent was obtained from all patients, and the study was approved by the Institutional Review Board of Changhai Hospital.

Finally 310 patients were enrolled in the clinical research. Diagnosis of CAS was confirmed based on the criteria of TOAST trial [11]. Patients included in the study either presented with focal neurological symptoms related to their anterior cerebral circulation (transient ischemic attack, amaurosis fugax, and stroke) within 6 weeks of surgery (further defined as symptomatic) or presented with no history of neurological symptoms (further defined as asymptomatic). All the symptoms were confirmed with radiological evidences provided by independent radiologists. The statuses of HP infection in patients were confirmed by the urea breath-test.

Evaluation of family history, hypertension, diabetes, cigarette intake and hyperlipidemia was also performed in both groups. The definition is as follows:

1. Hypertension: if they had diastolic blood pressure >90 mmHg and/or systolic blood pressure >140 mmHg or if they were chronically (>6 months from evaluation) taking anti-hypertensive drugs.
2. Diabetes: if they had fasting levels of glucose >7.0 mmol/L in two distinct instances or if they were chronically (>6 months from evaluation) taking hypoglycemic drugs.
3. Current smokers: if they were active smokers or their abstinence from smoking was <30 days at the enrolment visit.
4. Hypercholesterolemia: if subjects had levels of total serum cholesterol >220 mg/dl or they were chronically (>6 months from evaluation) taking lipid-lowering drugs.

### Serology in Patient

Blood samples were collected from all patients and controls and stored at −80°C until analysis. Sera of patients and controls were analyzed at the same time. Specific human anti-H. pylori IgG antibodies were detected by an enzyme-linked immunosorbent assay (ELISA) kit (Virion-Serion, Germany) according to the manufacturer's instructions. Values over 10 U/ml (sensitivity and specificity >95%) were defined as infections. In infected group, a serological assay for specific human IgG against CagA was also performed (Jingying Biotech, China). Titers were defined as positive or negative according to a cut-off value of 10 U/ml (sensitivity and specificity >95%). Serum YKL-40 and C-reaction protein (CRP) concentrations were determined by commercial ELISA kits.

### Animals and Treatment

Male New Zealand white rabbits, purchased from the animal center of Second Military Medical University, were bred and maintained there. This investigation was performed in strict accordance with the recommendations in the Guide for the Care and Use of Laboratory Animals of the National Institutes of Health. All the animal procedures were reviewed and approved by the Committee on the Ethics of Animal Experiments of the Second Military Medical University. All efforts were made to minimize sufferings of animals.

The rabbits were 8 weeks old at the time of entry into the study (n = 36). All the rabbits were placed on a high-cholesterol diet (containing 1% cholesterol, 5% axungia porci and 7.5% yolk powder) [12]. Water was provided ad libitum.12 weeks later, the rabbits were randomly divided into 2 groups (A = CagA^+^ HP infection; B = control). Group A was injected to intravenously with CagA^+^ HP strains.

In the 14^th^ week, blood samples of all rabbits were harvested from ear vein to measure serum YKL-40 and CRP. After that, 3 animals of each group were anesthetized with intramuscular injection of ketamine (50 mg/Kg) and dehydrobenzperidol (5 mg/Kg), and then they were sacrificed by air injection into the ear vein. The carotid arteries were removed and washed with icy saline. The carotid arteries were divided into three pieces at 5 mm intervals, fixed with 4% paraformaldehyde and embedded in paraffin. The samples were routinely stained with hematoxylin-eosin and oil red O for lipid visualization. The intima-media thickness (IMT) and plaque size were also measured. All images were taken under an Olympus microscope and analyzed by computer with Axioplan 2 imaging software (Zeiss, Germany).

Group A was further randomly divided into 2 subgroups. A1 (n = 7) animals were re-injected with CagA^+^ HP strains; A2 (n = 8) received classic anti-HP treatment (Omeprazole 6 mg+Amoxicillin 0.3 g+Clarithromycin 0.152 g, bid, 7days) instead. The plaque features in each group were demonstrated by ultrasound. 4 weeks later, all the rabbits were euthanized, and the serum YKL-40, CRP and plaque morphology were re-evaluated.

### Animal Carotid Color Doppler Ultrasound

Bilateral assessment of the carotid arteries was performed by a well-trained operator. The test was carried out with a Philips iE33 xMATRIX echocardiography system (Philips, Netherland) with a linear 7.0 MHz probe. The carotid trunk was identified using both B-mode and pulsed-wave color Doppler ultrasonography. Plaque was defined as a protrusion into the vessel lumen of at least 2 mm, as measured from the border between the adventitial and medial layers. The main characteristics of atherosclerotic carotid plaques (degree, surface characteristics) were evaluated.

Stage of lesion progression was assessed with Virmani’s classification criteria [13]. Of 6 defined categories (i.e. 1: fibrous lesion; 2: atheromatous lesion; 3: thin cap atheroma; 4: healed rupture; 5: plaque rupture or intraplaque hemorrhage, and 6: plaque erosion), the first 2 classes are considered stable, and the others are perceived as unstable plaques.

### Statistical Analysis

Continuous variables are expressed as mean ± SD, and categorical data are presented as percentages. Normality of the continuous variables was examined by the Kolmogorov–Smirnov test. A Fisher’s exact test or χ-test was performed for categorical variables. Differences in continuous variables between two groups were assessed by an unpaired 2-tailed t-test. The role of YKL-40 in predicting symptomatic CAS was assessed using Cox regression analysis. A 2-tailed p<0.05 was considered statistically significant for all the above analysis. All statistical analysis was performed with SPSS 11.0 for Windows (SPSS, Inc., Chicago, IL, USA).

## Results

### Clinical Characteristics

The study population included a total of 310 participants (196 men and 114 women) undergoing color Duplex assessment. And 168 patients with CAS (109 Males and 59 Females; mean age: 64.7±9.4) were involved in CAS group. The characteristics and the laboratory findings of the both groups are detailed in Table 1. No significant differences were found in age, gender, hypertension, hyperlipidemia, diabetes or smoking history between the two groups.

**Table 1:** Demographic features, classic risk factor prevalence rate in CAS patients and controls.

Characteristics	CAS(n = 168)	Control(n = 142)	P value
**Age (mean±SD), year**	64.7±9.4	62.9±7.2	0.063
**Males (n,%)**	109(65)	87(61)	0.589
**Hypertension (n,%)**	102(61)	87(61)	0.986
**Current cigarette smoke (n,%)**	94(56)	67(47)	0.154
**Diabetes (n,%)**	76(45)	65(46)	0.984
**Hypercholesterolemia (n,%)**	78(46)	53(37)	0.133

### Increased Serum YKL-40 and CRP Levels in CagA^+^ HP Infection

**Figure 1:**
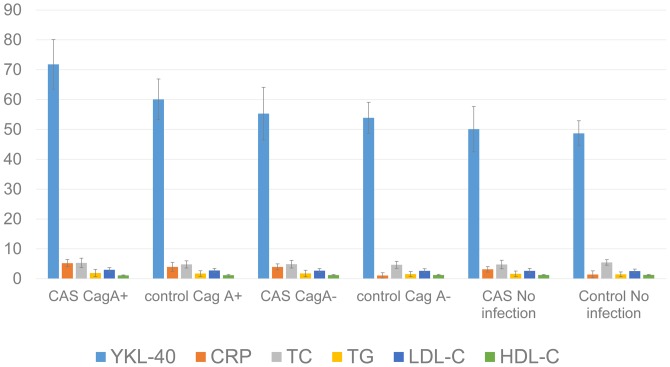
Serum biomarker and laboratory test in different subgroups. Serum YKL-40 level was significantly elevated in CagA+ HP infection group, and the levels of CRP, total cholesterol (TC), triacylglycerol (TG), low density lipoprotein cholesterol (LDL-C) and high density lipoprotein cholesterol (HDL-C) were not significantly different between groups.

All the 310 participants received blood sample analysis of HP infection and CagA+ HP infection. Seventy-six CAS patients and 31 non-CAS participants were confirmed to be infected with CagA+ HP, and 29 CAS patients and 24 non-CAS participants were confirmed to be CagA^-^ HP carriers. And the rest were free of infection. Compared with CagA^-^ HP infection group and controls, serum YKL-40 level in CagA^+^ HP infection group was significantly higher (P<0.0001), while CRP, a traditional inflammatory biomarker, was significantly higher in CAS group, regardless of the infectious status of HP (Figure 1). The level of total cholesterol, LDL and HDL cholesterol were also observed and no significant differences were found between the 2 groups.

### Increased Serum YKL-40 Level in Symptomatic CAS with CagA^+^ HP Infection

**Figure 2:**
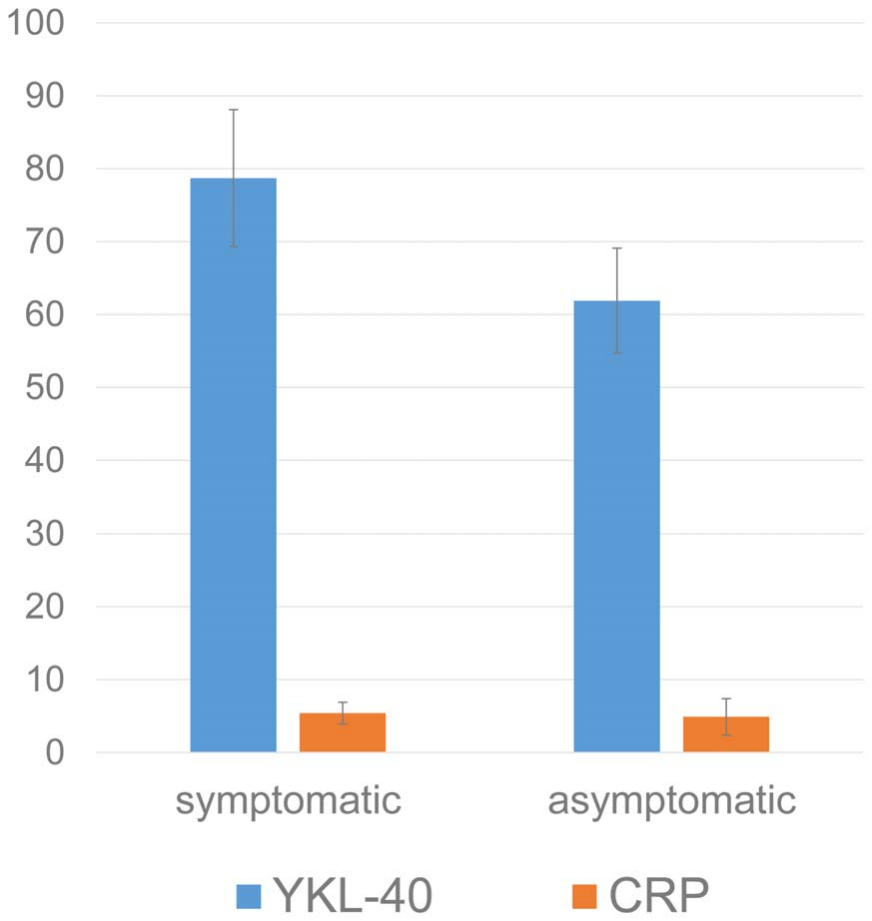
YKL-40 and CRP concentration stratified by symptoms. Serum YKL-40 and CRP levels in symptomatic or asymptomatic CAS with CagA^+^ HP infection. YKL-40 level increased significantly in the symptomatic group while CRP level showed no significant difference.

We regrouped 76 CAS patients with CagA^+^ HP infection into 2 groups according to the neurological symptoms. We evaluated the serum YKL-40 and CRP levels in both groups. Although increased in both groups, serum YKL-40 level was still significantly higher in the symptomatic group (P<0.0001) while CRP level showed no significant difference (P = 0.282) (Figure 2). Cox regression analysis demonstrated increase of YKL-40 was correlated with neurological symptoms (HR 2.280, 95% CI 1.584–4.287, P = 0.018), which confirming the role of YKL-40 in predicting poor clinical manifestation. This suggests that YKL-40 might be associated with symptom severity of CAS with CagA^+^ HP infection.

### Serum YKL-40 Level Increased along with Increased Plaque Instability and Increased IMT in the Animal Model of CAS with CagA^+^ HP Infection

**Figure 3:**
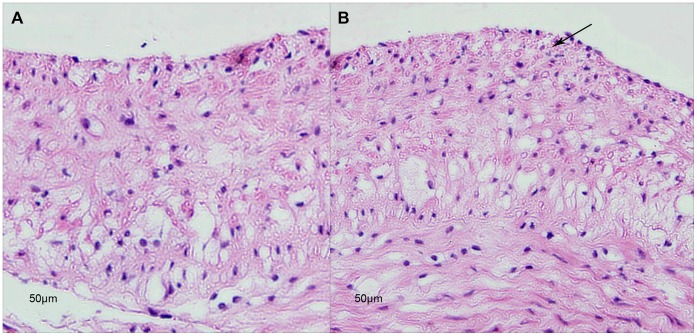
Hematoxylin and eosin stained cross sections of carotid artery in rabbits without CagA^+^ HP infection (A) and with CagA^+^ HP infection (B) at 14^th^ week. Arrow shows multi-layer of foam cells deposited.

Our clinical research revealed that, compared with the classic inflammatory biomarker CRP, serum YKL-40 level might be more specifically related to CAS with CagA^+^ HP infection and its clinical symptom severity. In order to confirm their relationship, we further created a CAS animal model with New Zealand white rabbits. After 12 weeks’ high-cholesterol diet on both groups and 2 weeks’ infection with CagA+ HP in Group A, we found significantly increased serum YKL-40 and CRP levels in the infected group (Table 2). Unstable carotid plaques were found in two thirds of rabbits in Group A by ultrasound examination. Average IMT was 0.7±0.1 mm in Group A, which was significantly thicker compared with 0.5±0.1 mm in Group B. Cross sections of carotid arteries stained with hematoxylin and eosin revealed that multi layers foam cells deposited in Group A (Figure 3).

**Table 2:** Plasma YKL-40, CRP level and plaque morphology in group A(hypercholesterolemia rabbits with CagA^+^ HP infection) and group B(hypercholesterolemia rabbits without CagA^+^ HP infection) at 14^th^ week.

Index	A(n = 18)	B(n = 18)	P value
**YKL-40 (ng/ml)**	12.6±1.1	5.8±0.5	<0.0001
**CRP (mg/L)**	153.6±35.7	132.8±18.5	0.035
**Unstable plaque (n, %)**	12(67%)	2(11%)	0.002
**IMT (mm)** *	0.7±0.1	0.5±0.1	<0.0001

* IMT were measured in one sixth of each group.

### Declined Serum YKL-40 Level Accompanied by anti-HP Therapy and Plaque Stabilization

**Figure 4:**
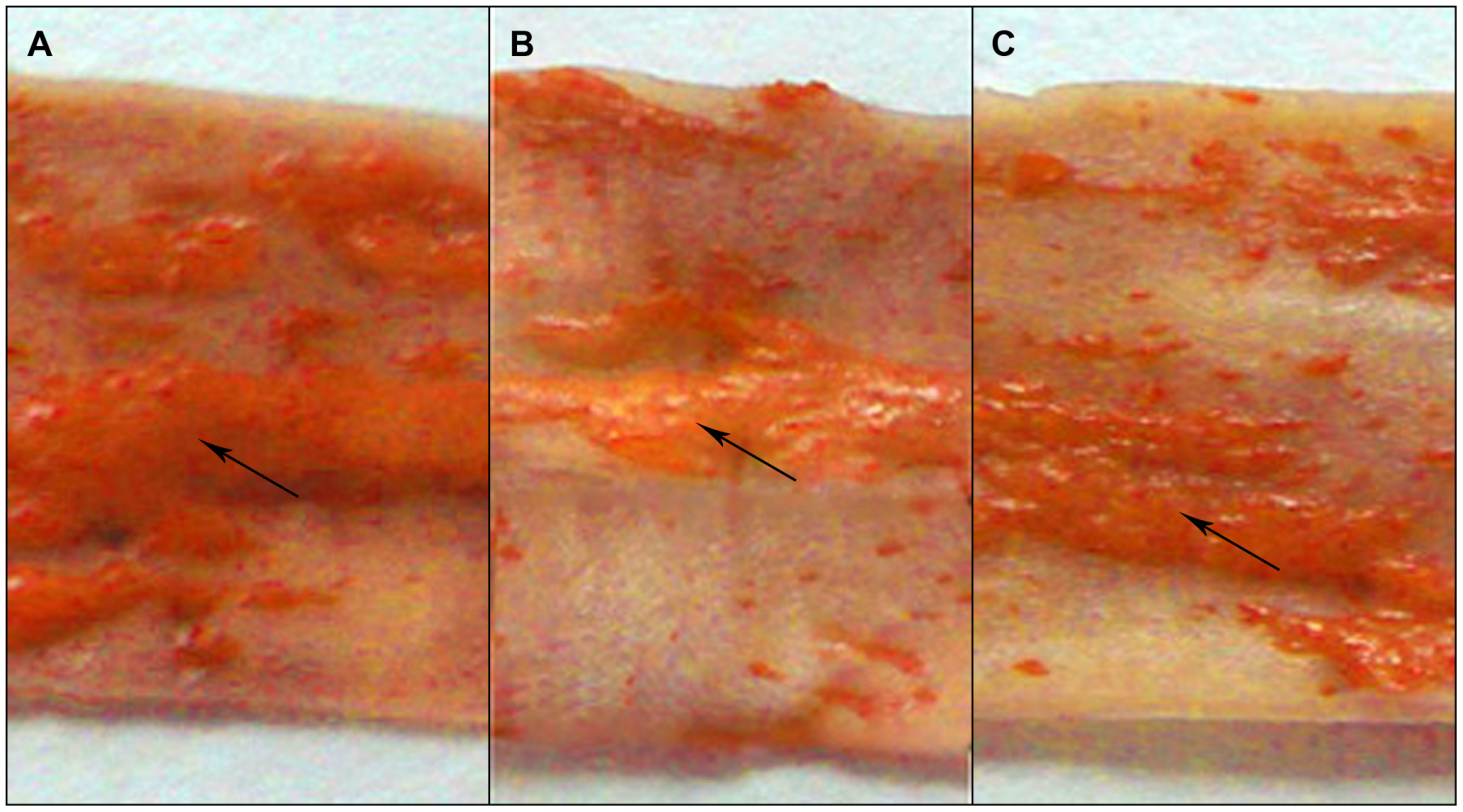
Carotid artery stained with oil red O lipid visualization in CagA^+^ HP infection group (A1), anti-HP infection treatment group (A2) and controls (B). Arrows show lipid deposition in carotid artery.

After 4 weeks’ anti-HP treatment in Group A2, we detected serum YKL-40 and CRP levels in those 3 groups along with plaque morphology. YKL-40 and CRP remained at a high level in Group A1, but YKL-40 decreased to 5.9±0.6 ng/ml in Group A2, with no significant difference when compared with that in Group B, while CRP maintained at a high level (Table 3). Although Group A2 seemed to have a higher unstable plaque ratio, half of the first 6 unstable plaques at the beginning of anti-HP treatment became stable at last. We also evaluated plaque stability under ultrasound and stained the specimen with oil red O for lipid visualization to measure plaque size (Figure 4).

**Table 3:** Analysis of plasma YKL-40, CRP level and plaque morphology in group A1, A2 and B at 18^th^ week.

	A1(n = 7)	A2(n = 8)	B(n = 15)
**YKL-40 (ng/ml)**	19.5±2.1* #	5.9±0.6†	5.8±0.5
**CRP (mg/L)**	191.6±44.8^△#^	187.5±32.5#	147.8±33.7
**Unstable plaque (n, %)**	6 (86%)* #	2 (25%)†	3 (20%)
**Plaque size (mm^2^)**	6.8±1.1* #	3.7±0.7†	3.9±0.9

* p<0.05, significantly different from group A2;

# p<0.05, significantly different from group B;

† p>0.05, no significantly different from group B.

△ p>0.05, no significantly different from group A2.

## Discussion

The relationship between CagA^+^ HP infection and atherosclerosis has been detected already, and this special infection has been demonstrated to be associated with atherosclerosis independently [1]. During a 5-year follow-up, the carotid artery IMT of patients infected with CagA^+^ HP has shown a remarkable increase compared with that of negative ones [14]. And recent studies have demonstrated that such kind of infection might cause vulnerable plaques [2], which lead to poor prognosis of related conditions.

Due to the high incidence of HP infection in China, especially CagA^+^ strains [15], investigation of such connection is of great significance in protecting CAS patients from ischemic stroke.

Inﬂammatory processes play a pivotal role in all stages of atherosclerosis, from the initial induction of endothelial dysfunction and plaque formation to plaque destabilization and disruption with superimposed thrombosis which leading to acute myocardial infarction or death [16]. During the entire process, there is a contribution from activated inﬂammatory cells such as neutrophils, lymphocytes, monocytes and resident macrophages, which secret pro-inﬂammatory cytokines. The classic inﬂammatory biomarker CRP has been evaluated as a prognostic biomarker in patients with ischemic heart disease with an acceptable sensitivity [17]. However, since it is mainly produced in the liver, the concentration may be interfered by many other factors, resulting in poor specificity. In our study, we focused on a special type of carotid atherosclerosis with CagA+ HP infection, which is common in China. So it is likely that novel biomarkers secreted by inﬂammatory cells within the atherosclerotic plaque could be superior prognostic biomarkers. The role of YKL-40 in atherosclerosis has been discussed previously, especially in coronary and peripheral arteries, but less investigated in the carotid artery.

In the present study, we first found that serum YKL-40 level in the group of CAS with CagA^+^ HP infection was significantly elevated compared with controls, but remained equal in the other two groups. We also found that CRP concentration was increased in all the 3 groups, which it failed to be a specific biomarker of CagA^+^ HP infection. Moreover, as a specific type of infection, CagA+ HP is confirmed to be a potential risk factor for symptomatic CAS, therefore a novel biomarker correlated with CagA+ HP is of great significance. We further detected the relationship between serum YKL-40 or CRP level and neurological symptoms. We found significantly increased YKL-40 concentration was associated with symptomatic CAS in the CagA^+^ HP infection group; and CRP, the classic inflammatory biomarker, failed to reveal such a quantitative relationship with CAS symptoms.

Our findings support the previous suggestion that inflammation-related increase of YKL-40 is associated with atherogenesis and carotid atherosclerosis progression. Whether YKL-40 is an active player or just an innocent bystander is not clear. We believe that the relationship between YKL-40 level and atherosclerosis may represent a new opportunity for the possible utility of serum YKL-40 as an inflammatory marker for carotid atherosclerosis.

To confirm the predictive ability of YKL-40 in carotid atherosclerosis with CagA^+^ HP infection, we created a CAS animal model with New Zealand white rabbits. Increased serum YKL-40 level was found to be associated with CagA^+^ HP infection, and accompanied by more unstable plaques and increased IMTs. After receiving anti-HP therapy, serum YKL-40 level declined to the normal level, followed by more stable plaques and smaller plaque area. Based on such findings, we confirmed the specific relationship between YKL-40 and CAS with CagA^+^ HP infection. Also we concluded that overexpression of serum YKL-40 level might reveal the unstable status of carotid plaque.

To our acknowledge, no other centers have evaluated the YK-40 in CagA+ HP infection. As a new inﬂammatory biomarker, YKL-40 may be a potential circulating biomarker of interest in the risk assessment and curative effect evaluation in CAS patients with CagA^+^ HP infection. However, to be a new biomarker, three fundamental requirements have to be further discussed: (1) assay must be accessible to clinicians and analytical methods must be accurate and reproducible; (2) it must provide new prognostic information in multiple studies and diverse populations, with incremental prognostic information when added to validated risk scores; and (3) there must be evidence that associated risk is modifiable with specific therapy or in other way enhances care [18].

### Conclusion

In summary, our results indicate that YKL-40 is much higher in CAS patients with CagA^+^ HP infection, and it may be an atherogenic serum biomarker. YKL-40 overexpression can predict vulnerable plaques in rabbits. Our findings suggest the specificity of YKL-40 in atherosclerosis, especially in patients suffering from CagA^+^ HP infection, indicating that YKL-40 may serve as a predictive biomarker for plaque instability in carotid atherosclerosis with CagA^+^ HP infection.
